# Plant-Derived Dipeptidyl Peptidase-4 Inhibitors in Cardiometabolic Disorders: A Critical Review of Natural Scaffolds and Experimental Evidence

**DOI:** 10.3390/biomedicines14102141

**Published:** 2026-09-22

**Authors:** Kuanysh Zhapar, Diana Samatkyzy, Diana Valishayeva, Saule Rakhimova, Dos Sarbassov, Ainur Akilzhanova

**Affiliations:** 1 Centre for Life Sciences, National Laboratory Astana, Nazarbayev University, Kabanbay Batyr Avenue 53, Astana 010000, Kazakhstandiana.valishayeva@nu.edu.kz (D.V.); saule.rakhimova@nu.edu.kz (S.R.);; 2 Department of Biological and Biomedical Sciences, School of Medicine, Nazarbayev University, Kabanbay Batyr Avenue 53, Astana 010000, Kazakhstan

**Keywords:** DPP-4, dipeptidyl peptidase-4, plant-derived inhibitors, natural products, chalcones, aurones, flavonoids, cardiometabolic disorders, type 2 diabetes mellitus

## Abstract

Cardiometabolic disorders, including obesity, insulin resistance and type 2 diabetes mellitus, remain major biomedical problems closely linked to vascular complications. Dipeptidyl peptidase-4 (DPP-4) is a clinically validated therapeutic target because inhibition of this enzyme prolongs the activity of incretin hormones and supports glucose-dependent insulin secretion. This review compiles and critically evaluates plant-derived DPP-4 inhibitors, with emphasis on chemical class, enzyme source, quantitative activity, assay interpretability, and the translational relevance of reported findings. The available literature indicates that flavonoids and polyphenolic compounds dominate the field, although assay heterogeneity, medium effects, potential aggregation, and insufficient selectivity data limit interpretation. Among natural scaffolds, chalcones and aurones, including lanceoletin, leptosidin, xanthoangelol, chalconaringenin 2′-O-β-D-glucopyranoside, and aureusidin 6-O-β-D-glucopyranoside, represent comparatively well-supported lead-like scaffolds that warrant further standardized evaluation. The evidence does not support using plant-derived DPP-4 inhibitors as substitutes for approved antidiabetic drugs. Their greatest value lies in expanding chemical space and providing lead-like scaffolds for standardized potency, selectivity, kinetic and preclinical validation.

## 1. Introduction: Cardiometabolic Disorders and Current Status of the Issue

Cardiometabolic disorders constitute a continuum of disorders where obesity, insulin resistance, Type 2 Diabetes Mellitus (T2DM), dyslipidemia, hypertension and cardiovascular complications mutually reinforce each other. In the current literature, insulin resistance has been identified as the most significant link connecting all changes in metabolism with the progression of vascular damage and an increase in the risk of cardiovascular complications [1,2].

A particularly unfavorable version of this continuum occurs with visceral obesity, when fat tissue acquires characteristics of an active endocrine and pro-inflammatory organ. Under these conditions, chronic inflammation, lipotoxicity, insulin signaling defectiveness and compensatory hyperinsulinemia develop into beta cell function loss and persistent hyperglycemia. Hyperglycemia creates oxidative stress, non-enzymatic glycation of proteins, endothelial dysfunction and microcirculation defects. Thus, T2DM can be viewed both as a metabolic disorder and as a vascular disorder [2,3].

## 2. DPP-4 as a Therapeutic Target: Biological Function and Mode of Action of DPP-4 Inhibitors

DPP-4 is a membrane-associated and soluble serine protease that rapidly inactivates several bioactive peptides, including the incretin hormones glucagon-like peptide-1 (GLP-1) and glucose-dependent insulinotropic polypeptide (GIP). By cleaving these substrates, DPP-4 limits the duration of incretin signaling and thereby contributes to the regulation of postprandial glucose metabolism (Figure 1). Pharmacological inhibition of DPP-4 prolongs endogenous incretin activity, enhances glucose-dependent insulin secretion, and suppresses glucagon release, providing the mechanistic basis for the clinical use of DPP-4 inhibitors in T2DM [4,5,6]. This clinically validated mechanism provides the reference framework for assessing whether plant-derived compounds demonstrate reproducible and pharmacologically meaningful DPP-4 inhibition.

## 3. Clinical Use of DPP-4 Inhibitors

Clinically approved DPP-4 inhibitors are orally administered, weight-neutral glucose-lowering agents associated with a low risk of hypoglycemia and moderate reductions in HbA1c. Cardiovascular outcome trials generally support the cardiovascular safety of this drug class, although agent-specific differences have been reported, including an increased risk of hospitalization for heart failure with saxagliptin [7,8,9,10,11]. Their established clinical use validates DPP-4 as a pharmacological target and provides clinically relevant reference comparators for evaluating plant-derived inhibitors.

As summarized in Table 1, multiple individual DPP-4 inhibitors have entered clinical use, confirming the sustained pharmacological validation of this therapeutic class. Table 2 further shows that gliptins have been incorporated into fixed-dose combinations with metformin, sodium–glucose cotransporter-2 inhibitors, and pioglitazone, thereby extending their use within multidrug treatment strategies. Collectively, the single-agent and combination products presented in Table 1 and Table 2 confirm DPP-4 as a clinically validated therapeutic target. In the context of this review, these agents serve as reference standards, whereas plant-derived inhibitors should be regarded primarily as starting scaffolds for further optimization rather than as alternatives to approved therapies.

## 4. Natural Compounds as Sources of New DPP-4 Inhibitor Scaffolds

Natural compounds could potentially become a rich source of novel DPP-4 inhibitors. There is little to no interest in replacing existing drugs based on natural compounds. Nonetheless, these compounds can serve as useful resources for discovering new scaffolding possibilities, especially when they exhibit pharmacophores and/or lead-likeness. As such, natural products continue to play a very significant role in drug-discovery efforts, particularly due to the structural diversity associated with these types of compounds. Therefore, they remain a valuable tool for the identification of additional pharmacophore-, lead- and scaffold-like scaffolds [32,33].

Given that there is now a clinically validated class of gliptin-based DPP-4 inhibitors currently available, finding new scaffolds continues to be a worthwhile pursuit. Given the limitations of some of the gliptin candidates in terms of efficacy, selectivity and translatability into humans, natural product-derived molecules should therefore primarily be viewed as suitable starting points for subsequent medicinal chemistry optimization. Ultimately, viewing plant-derived molecules as potential chemical starting points for further medicinal chemistry investigation may help to prevent an overinterpretation of in vitro signal generation and may assist in distinguishing promising molecular leads from premature therapeutic claims [32,33].

## 5. Objective of the Review

The aim of this structured critical review is to synthesize the existing knowledge regarding plant-derived DPP-4 inhibitors while critically evaluating the strength and interpretability of the experimental evidence. The review compares major chemical classes, examines structure–activity relationships, and identifies molecular scaffolds that warrant further pharmacological investigation. Particular attention is given to heterogeneity in enzyme sources, assay conditions, compound characterization, mechanistic evidence, selectivity, and potential assay interference [34,35].

Therefore, the objectives of this review are:

(1) Structured compilation of information reported on plant-derived DPP-4 inhibitors by chemical class.
(2) Analysis of structure–activity relationships.
(3) Evaluation of the quality/validity of experimental data as it relates to the form/type of material being tested; source of enzyme utilized; and whether or not specific quantitative parameters are presented.
(4) Identification of those compounds exhibiting the highest degree of promise as initial chemical entities for further development towards producing new DPP-4 inhibitors. In conducting this review, considerable emphasis has been placed upon those studies employing single compounds; a clearly defined enzyme system; and whenever possible selectivity data since these studies are generally more translatable.

### Search Strategy and Criteria for Exclusion and Prioritization

This article was conducted as a structured critical review rather than a formally registered systematic review. The literature search was conducted from 5 January to 25 February 2026. Literature searches were performed in PubMed, Scopus, Web of Science, ScienceDirect, and Google Scholar, with supplementary discovery through reference-list screening and conference proceedings. The core search concepts combined terms related to “DPP-4”, “DPP-IV”, “dipeptidyl peptidase-4” or “dipeptidyl peptidase IV” with terms for plant-derived compounds, natural products, flavonoids, chalcones, aurones, alkaloids, phenolics, and enzyme inhibition. Peer-reviewed original studies reporting experimental DPP-4 inhibitory activity of individual plant-derived compounds or clearly characterized bioactive molecules were prioritized. Reviews, editorials, opinion papers, reports without experimental DPP-4 data, and studies not involving plant-derived compounds were excluded from the primary evidence synthesis. During data extraction, compound identity, botanical source, DPP-4 source, reported IC_50_ or inhibition data, kinetic or mechanistic information, selectivity data where available, and evidence relevant to assay interference were recorded. According to the literature-selection dataset summarized in Figure 2, 1276 records were identified, 1176 records remained after duplicates were removed and were screened, 284 full-text articles were assessed for eligibility, and 78 studies were included in the review. Because enzyme sources, substrates, assay formats, and reporting practices were highly heterogeneous, no meta-analysis was undertaken. Figure 2 summarizes the literature identification, screening, eligibility assessment, and final study-selection process for transparency; the use of a flow diagram does not imply classification of this article as a systematic review.

## 6. Plant-Derived DPP-4 Inhibitors: Structured Compilation and Critical Appraisal of Experimental Evidence

The existing literature detailing the biological activities exhibited by plant-derived molecules as DPP-4 inhibitors exhibits both a high rate of publication growth and considerable methodological heterogeneity. Existing studies present together data derived from evaluations of individual compounds; partially characterized fractions; and crude extracts containing multiple bioactive components. Further complicating comparison between studies are differences in enzyme source utilization; assay formats employed; and depth of mechanistic understanding provided for each study’s findings. Consequently, detecting activity alone does not permit equal valuation for future development purposes among all molecules investigated. Amongst studies reporting DPP-4 inhibitory activity for an individual compound, those demonstrating activity against a clearly defined system (preferably utilizing human recombinant DPP-4) coupled with a minimal level of mechanistic insight into enzyme–inhibitor interactions represent the most informative [35,36].

An important interpretive caveat applies particularly to polyphenol-rich DPP-4 inhibitor studies. Many phenolic compounds may display behavior associated with pan-assay interference compounds (PAINS), generating apparent activity through nonspecific mechanisms such as colloidal aggregation, optical interference, redox activity, and extensive protein binding [37,38]. Importantly, a PAINS structural alert and experimentally demonstrated aggregation are not synonymous; such alerts should prompt confirmatory testing rather than automatic exclusion. Pohjala and Tammela [37] showed that a subset of phenolic compounds formed detectable aggregates at concentrations as low as 10 µM, while Proença et al. [34] demonstrated that flavonoid-mediated DPP-4 inhibition can be markedly attenuated in protein-rich environments. Consequently, positive results for flavonoids, tannins, and other polyphenols should be interpreted cautiously unless supported by appropriate controls, detergent-sensitivity testing, or orthogonal assay formats [34,37,38].

### 6.1. Flavonoids

Flavonoids continue to be the most researched class of natural dipeptidyl peptidase-4 (DPP-4) inhibitors. The large amount of accumulated data regarding this class allows us not only to consider the potential DPP-4-inhibiting capacity of each compound but also the first structure–activity correlations. For example, Pan et al. have shown that the ability of flavonoids to inhibit DPP-4 is influenced by several minor variations in terms of the degree of hydroxylation and substitutions at the ring positions. Furthermore, certain structural changes within the flavonoids can either enhance or diminish their affinity towards the DPP-4 enzyme. Despite the fact that Pan et al. identified clear structural patterns for flavonoids, they noted that due to the use of different test systems, substrates, and experimental conditions among various investigations, comparison of results from these studies was greatly complicated [35].

Practically speaking, it is not the flavonoid class as a whole, but rather a series of individual compounds that demonstrate inhibitory activity in a defined system. An example would be flavonol-glycosides extracted from *Lens culinaris* seeds for which Kim et al. found IC_50_ values of 27.89, 36.52 and 37.01 μM against human recombinant DPP-4. Although these compounds do not present the highest potencies compared to those previously reported, they possess great value as a consistently defined series for SAR analysis purposes and provide a clean and effective methodological approach for assessing the DPP-4 inhibitory activity of natural compounds [39]. Accordingly, reported flavonoid DPP-4 inhibition should be interpreted in the context of possible aggregation and matrix-dependent effects, particularly when orthogonal validation is absent [34,37].

### 6.2. Phenolic Acids and Other Simple Phenolic Structures

Unlike flavonoids, the investigation of phenolic acids and other simple phenolic structures represents a much less systematic field of inquiry. As such, many studies concerning these types of compounds report evidence of inhibitory activities against DPP-4; however, in addition to the variability in testing methods utilized to evaluate the activity of these compounds, results often depend heavily upon the testing environment used. In addition to reporting the potential inhibitory activity of individual compounds, it is common for studies involving these types of compounds to evaluate them as part of crude extracts prepared from plants. Due to the complexity associated with distinguishing between true enzymatic inhibition and matrix-related analytical artifacts when utilizing assay formats that include crude plant extracts or similar matrices, the use of phenolic acids in research continues to represent a secondary or auxiliary area of research relative to identifying lead compounds [35,36].

Although individual phenolic and phenylpropanoid derivatives undoubtedly warrant consideration based upon their contribution to chemical diversity, it appears that their value lies more in extending the scope of compounds being evaluated versus establishing a top-tier priority list of candidate compounds for immediate medicinal chemistry-based optimization. The use of human recombinant DPP-4, together with the standardization of all assay parameters, would generate more comparable data and enable a more definitive assessment of these compounds [35]. For example, methyl p-coumarate from *Melicope latifolia* and diferuloylsucrose derivatives from *Lilium longiflorum* have shown measurable DPP-4 inhibition, but they remain biochemical screening leads rather than validated candidates [40,41].

### 6.3. Tannins, Proanthocyanidins, and Complex Polyphenolic Mixtures

Many studies have reported high levels of DPP-4 inhibitory activity for tannins/proanthocyanidin mixtures/complex polyphenolic structures. This is precisely one of the groups where reliable interpretation of results is greatly complicated by interactions exhibited by high-molecular-weight polyphenols during DPP-4 enzyme assays. Interactions between the high-molecular-weight polyphenols and proteins in the media surrounding the cells can limit the ability to draw meaningful comparisons between studies. Proença et al. demonstrated that the inhibitory effect exhibited by flavonoids against DPP-4 is severely diminished when tested in a protein-rich media. This finding has broader implications for all polyphenolic complexes as an entire class: the closer an assay is to mimicking a biological environment, the more caution should be exercised when interpreting a positive result achieved using a simplified assay [34].

Therefore, results obtained from studies investigating tannins/proanthocyanidins should generally be viewed as indicative of potentially useful chemical motifs, rather than directly contributing to an ordered ranking of lead compounds. Further development of this group should focus on compositional deconstruction, isolation of individual compounds exhibiting activity, and validation of activity using more stringent assay formats [34,36].

This concern is particularly relevant to tannins and proanthocyanidins, whose high molecular weight, extensive hydrogen-bonding capacity, and strong protein-binding properties may complicate interpretation of apparent enzyme inhibition. Positive results from simplified biochemical assays should therefore be treated as screening evidence rather than definitive proof of target-specific DPP-4 inhibition [34,37]. The reported inhibition of DPP-4 by the ellagitannins rugosin A and rugosin B from *Rosa gallica* should be interpreted within this same assay-interference framework [42].

### 6.4. Alkaloids

Alkaloids represent a chemically distinct, non-polyphenolic class that expands the search space beyond recurrent flavonoid motifs. Berberine is the most prominent representative in the current literature. Al-Masri et al. [43] reported in vitro DPP-4 inhibition by berberine, while molecular docking in that study proposed a plausible orientation within the catalytic region of the enzyme. However, docking should be interpreted as hypothesis-generating computational evidence and does not by itself establish residue-specific binding or experimental target engagement. Berberine therefore remains useful primarily as a chemically distinct alkaloid scaffold for further validation and optimization [43,44].

Scaffolds derived from alkaloids are particularly attractive for additional medicinal chemistry work because they open up possibilities for developing selective binding and better pharmacokinetics by avoiding recurring polyphenolic motifs. Therefore, a few good examples of alkaloids could be extremely useful for discovering new DPP-4 inhibitors [36,43].

### 6.5. Chalcones, Aurones, and Chemically Attractive Leads

Within the available literature on natural DPP-4 inhibitors, chalcone and aurone derivatives are among the more consistently supported lead-like scaffolds for further investigation. Lanceoletin, Leptosidin and closely related analogues were identified by Kim et al. [45] in *Coreopsis lanceolata* using a human recombinant DPP-4 system. The three most active compounds had IC_50_ values of 9.6, 13.3, and 14.3 µM. While a few compounds exhibited stronger potency, many others showed weaker inhibitory effects. What makes this series attractive is that they combine meaningful quantitative activity with chemically tractable scaffolds that could be easily modified into additional analogues. This characteristic is crucial for creating large libraries of compounds to test against biological targets.

The evidence from *Coreopsis lanceolata* is further supported by an independent series of compounds isolated from the botanically distinct species *Helichrysum arenarium*. This second series is particularly informative because it provides independent biochemical support for recurring chalcone and aurone chemotypes. Morikawa et al. [46] isolated chalconaringenin 2′-O-β-D-glucopyranoside, aureusidin 6-O-β-D-glucopyranoside, and related molecules with DPP-4 inhibitory activity. Although their IC_50_ values were higher than those of the most active Coreopsis compounds, the study confirmed that both chalcone and aurone motifs can recur as active structures across distinct botanical sources. Importantly, kinetic analysis showed mixed-type inhibition for chalconaringenin 2′-O-β-D-glucopyranoside and aureusidin 6-O-β-D-glucopyranoside [46].

Collectively, these studies indicate that chalcone and aurone scaffolds have comparatively strong biochemical support within the current literature, although their translational potential remains insufficiently established.

### 6.6. Terpenoids and Other Less Common Classes

While individual studies indicate that natural compounds outside of the flavonoid and alkaloid families can inhibit DPP-4 activity, fewer good quality examples exist in these categories. Individual results in these groups will remain questionable until sufficient characterization of the enzyme system used occurs along with repeated validations and additional experimental evidence demonstrating consistency across multiple assays.

A review of the literature clearly indicates that while the catalog of natural DPP-4 inhibitors represents a useful resource for scientists studying DPP-4 inhibitors, its primary utility lies not in generating lists of “active” molecules but rather in sorting the literature data according to how suitable each piece of literature is for subsequent optimization efforts. Specifically, several chalcone-based series and aurone series stand out as high priorities for future work, as do certain flavonol glycoside series for SAR analysis and some alkaloid-based scaffolds, specifically those derived from the berberine framework. On the other hand, data on uncharacterized extract systems and polyphenolic systems lacking adequate characterization and weak comparative activity data will be viewed as preliminary and requiring subsequent verification.

## 7. Discussion

### 7.1. Structure–Activity Relationships and Molecular Interactions with DPP-4

Pan et al. [35] systematically evaluated 70 structurally diverse flavonoids, providing the most coherent experimental basis for SAR interpretation in this literature. Hydroxylation at C3′ and C4′ of ring B and at C6 of ring A generally favored DPP-4 inhibition, whereas hydroxylation at C3 of ring C was often unfavorable. O-methylation at C3′, C4′, or C7 tended to reduce activity. The conjugated C2=C3 double bond together with the C4 carbonyl helped preserve a comparatively planar scaffold and was associated with stronger inhibition in matched flavonoid pairs. These relationships are summarized in Figure 3A.

These trends were not absolute. In the quercetin series, C3 O-methylation could improve activity, whereas methylation at C3′, C4′, or C7 generally had the opposite effect. Glycosylation also produced divergent outcomes: bulky sugar substitution could reduce binding through steric effects, but additional hydroxyl groups sometimes strengthened the apparent interaction, depending on the aglycone, sugar identity, and substitution position. Accordingly, these findings are best interpreted as within-series SAR patterns rather than universal rules or a basis for ranking IC50 values obtained under different assay conditions [35].

At the molecular level, the DPP-4 active site comprises the catalytic triad Ser630–Asp708–His740 and several ligand-recognition regions, including the hydrophobic S1 pocket, the negatively charged S2 pocket, and the S2-extensive region. The S1 pocket is formed predominantly by Tyr631, Val656, Trp659, Tyr662, Tyr666, and Val711, whereas Arg125, Glu205, Glu206, and Tyr662 contribute to ligand recognition in the S2 region. Phe357 and Arg358 participate in the S2-extensive region and can stabilize ligands with extended aromatic systems [47]. This architecture is relevant to chalcone and aurone scaffolds because their conjugated aromatic systems may support hydrophobic and aromatic interactions, while phenolic hydroxyl and carbonyl groups may participate in hydrogen bonding. These general structural considerations should nevertheless be distinguished from compound-specific docking evidence.

Published molecular-interaction data are currently strongest for xanthoangelol. Aulifa et al. [48] reported in vitro inhibition of human DPP-4 with an IC_50_ value of 10.49 µM. Their docking model predicted hydrogen bonds with the S2 residues Glu205 and Glu206 and a π–π interaction with Phe357 in the S2-extensive region, with a calculated binding energy of −8.34 kcal/mol and an estimated Ki of 0.873 µM (Figure 3B). These contacts suggest stabilization through the S2 and S2-extensive regions; however, a direct interaction with the catalytic Ser630 residue was not reported. The difference between the predicted Ki and the experimentally determined IC_50_ also illustrates that docking-derived affinity estimates should not be interpreted as quantitative substitutes for biochemical measurements. Accordingly, the reported pose provides a plausible binding hypothesis but does not establish residue-specific binding, target engagement, or the experimental mechanism of inhibition.

By contrast, the primary *Coreopsis lanceolata* study demonstrated biochemical DPP-4 inhibition by lanceoletin and leptosidin but did not report compound-specific molecular docking [45]. Their conjugated chalcone and aurone frameworks and multiple hydroxyl substituents are structurally compatible with hydrophobic-pocket occupancy and polar anchoring; however, specific interactions with Glu205, Glu206, Phe357, Ser630, or other active-site residues remain hypothetical. Dedicated docking followed by molecular dynamics simulations, site-directed mutagenesis, or structural studies would therefore be required to validate their proposed binding modes.

Within the same experimental series, comparisons are more informative. In *Coreopsis lanceolata*, lanceoletin (IC_50_ 9.6 µM), leptosidin (13.3 µM), 3,2′-dihydroxy-4,3′-dimethoxychalcone-4′-glucoside (14.3 µM), and 4-methoxylanceoletin (21.6 µM) were evaluated in the same human recombinant DPP-4 system [45]. In *Helichrysum arenarium*, chalconaringenin 2′-O-β-D-glucopyranoside (23.1 µM) was more active than the corresponding diglycoside (70.0 µM), illustrating that additional glycosylation can reduce activity within a defined series [46]. Such within-study comparisons provide a more defensible basis for SAR interpretation than cross-study ranking of absolute IC_50_ values.

**Figure 3:**
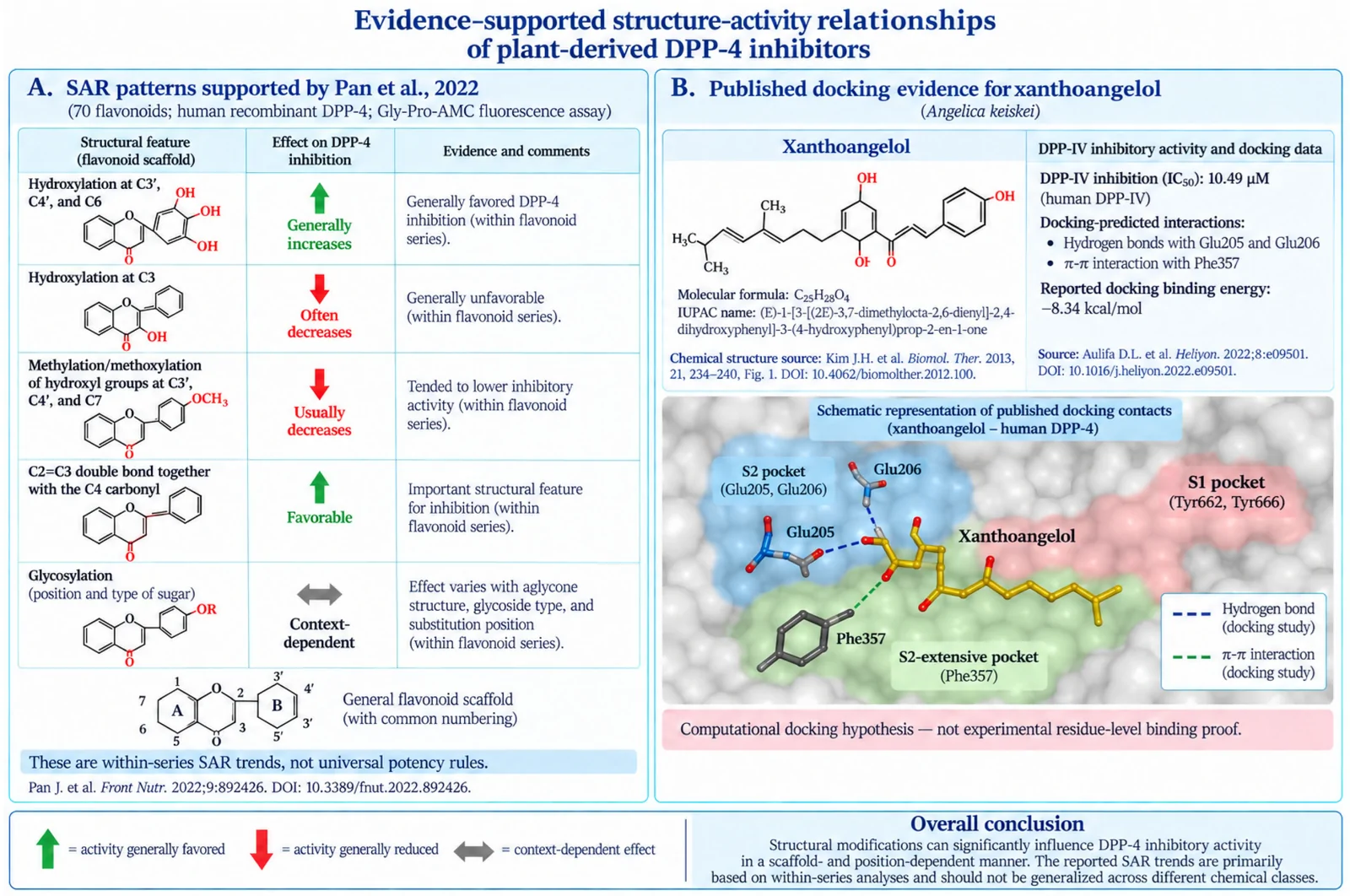
Evidence-supported structure–activity relationships of plant-derived DPP-4 inhibitors. (**A**) Within-series structure–activity trends for flavonoids reported by Pan et al. [35]. (**B**) Experimental DPP-4 inhibition and docking-predicted interactions of xanthoangelol reported by Aulifa et al. [48]. The chemical structure of xanthoangelol was redrawn based on Kim et al. [49]. Docking contacts and binding energy represent computational predictions and do not constitute experimental proof of residue-specific binding.

### 7.2. Reliably Interpretable Evidence and Data Requiring Careful Interpretation

The most reliable studies were conducted in a clearly identifiable enzymatic system with known DPP-4 origin; results were reported with quantitative detail. Such studies allow for comparison among different compounds and provide a basis for evaluating their potential for further investigation. The best available literature reports utilize human recombinant DPP-4 (*Coreopsis lanceolata* and *Lens culinaris*), and a few specific mechanistic examples (e.g., berberine). Although additional value is provided by reports detailing the nature of the interaction(s) between compounds and the target enzyme (i.e., whether reversible competitive or noncompetitive inhibition occurs), these studies offer a more informed level of interpretation regarding potential therapeutic utility.

On the other hand, data derived from extracts, poorly characterized fractions or inadequately documented assay protocols warrant careful interpretation. Polyphenolic systems present a unique problem since the bioactivity attributed to them may vary based upon the components within the medium being used. Proença et al. [34] demonstrated that the DPP-4 inhibitory effects of flavonoids in a protein-rich environment are substantially less effective than when assayed under more basic conditions. Consequently, high activity in a basic in vitro system does not necessarily indicate similar translatable relevance, nor does it justify advancement toward lead status for any compound.

Another critical parameter is specificity/selectivity. While demonstrating inhibition of the desired target enzyme (DPP-4) is a prerequisite for developing any DPP-4 inhibitor, demonstrating that this action is selectively directed towards DPP-4 vs. other members of the same enzyme family is equally essential. Lack of selectivity data combined with poor kinetic characterization remain two major shortcomings in the scientific literature concerning natural candidate DPP-4 inhibitors. Compounds with high IC_50_ values should be considered as preliminary leads until their profile has been evaluated in a more stringent comparative experimental format.

For critical comparison, the evidence can be viewed as a simple four-level hierarchy: Tier 1, biochemical DPP-4 inhibition only; Tier 2, biochemical inhibition supported by kinetic, mechanistic, or orthogonal evidence; Tier 3, biochemical evidence plus validation in cellular or more physiologically relevant systems; and Tier 4, evidence extending to in vivo activity together with selectivity and/or pharmacokinetic characterization. Most plant-derived compounds in the present literature remain at Tiers 1–2, which limits direct translational inference.

### 7.3. Priority Lead-like Scaffolds for Further Investigation

Prioritization was based on an integrated evidence assessment rather than on reported IC_50_ values alone, because direct potency comparisons across studies are confounded by differences in enzyme source, substrate, assay format, and experimental conditions. The main criteria were potency within a defined assay system, use of an individual chemically characterized compound, clarity of the DPP-4 source, availability of kinetic or molecular-interaction evidence, reproducibility within a structural series, and medicinal-chemistry tractability. Selectivity toward DPP-8/DPP-9 and cellular or in vivo validation were considered important strengthening factors when available, while their absence was treated as a limitation. On this basis, lanceoletin, leptosidin, xanthoangelol, chalconaringenin 2′-O-β-D-glucopyranoside, and aureusidin 6-O-β-D-glucopyranoside emerge as priority lead-like scaffolds for further investigation rather than as a definitive potency ranking across studies.

Importantly, the prioritization of these five compounds does not imply that they currently possess complete preclinical evidence or superior overall pharmacological profiles. Their selection reflects the relative strength and interpretability of the available evidence within the highly heterogeneous literature. In particular, these compounds were evaluated as chemically defined molecules in clearly described DPP-4 assay systems and were supported, to varying degrees, by within-series structure–activity relationships, kinetic characterization, or molecular-interaction data. Conversely, the limited availability of DPP-8/DPP-9 selectivity data, cellular validation, in vivo confirmation, and pharmacokinetic characterization remains a major limitation and prevents their interpretation as validated therapeutic candidates. Therefore, they are prioritized as lead-like scaffolds for further investigation rather than as established drug candidates.

From a translational perspective, however, none of the five prioritized compounds can currently be considered an advanced preclinical candidate. Lanceoletin, leptosidin, and xanthoangelol remain predominantly enzyme-stage leads because compound-specific cellular activity, in vivo DPP-4 target engagement, bioavailability, stability, metabolic fate, and pharmacokinetic exposure have not been established in the cited primary studies [45,48]. Chalconaringenin 2′-O-β-D-glucopyranoside and aureusidin 6-O-β-D-glucopyranoside have additional support from experimental kinetic analyses; however, the reported antihyperglycemic activity was demonstrated for the parent *Helichrysum arenarium* extract rather than for the isolated compounds [46]. Such extract-level activity cannot establish compound-specific efficacy, systemic exposure, metabolism, or DPP-4 target engagement in vivo. Consequently, the present prioritization reflects biochemical activity, evidence quality, and scaffold potential rather than translational maturity.

Lanceoletin exhibited low-micromolar activity with an IC_50_ value of 9.6 μM; therefore, it represents a potentially interesting natural lead compound. However, lanceoletin was significantly less active in comparison to sitagliptin (an IC_50_ value of 0.071 μM) in the same assay system. This difference illustrates how immature the development of this scaffold is at this time compared to other leads [45].

Berberine is another scaffold that is worthy of particular attention. Although it belongs to a class of compounds different from the polyphenols and lacks a large number of derivatives in the current DPP-4 literature, its significance derives from a different aspect. Berberine exemplifies the possibility of discovering effective natural DPP-4 inhibitors outside of flavonoid chemistry. Therefore, it serves as a useful reference for searching among alkaloids and broadens the chemical search space. Strategically, this is significant since expanding the chemical space increases the probability of identifying compounds that will have better selectivity profiles and better physical properties for optimization. However, berberine’s potential as an unmodified natural candidate is limited by its very poor oral bioavailability [44].

Even the strongest natural candidates remain lead scaffolds rather than final drug products. Further development requires standardized activity and kinetic testing, DPP-8/DPP-9 selectivity assessment, and progression to relevant cellular and preclinical models (Table 3 and Table 4).

Because IC50 is an assay-dependent parameter, values reported in different studies were not treated as directly comparable measures of intrinsic compound potency. Differences in enzyme origin, substrate, buffer composition, incubation conditions, detection method, and data-fitting procedures can substantially influence the reported IC50. Accordingly, cross-study IC50 values in Table 3 and Table 4 are presented descriptively rather than as a potency ranking. Quantitative potency comparisons and SAR interpretations were preferentially restricted to compounds evaluated within the same study and under the same experimental protocol [34,35].

Table 3 presents a concise set of representative compounds selected to illustrate the principal chemical classes, within-series SAR relationships, and differences in experimental context, including the DPP-4 enzyme source and assay system used in the cited primary study. The complete 39-compound catalogue is provided as Supplementary Table S1.

### 7.4. Selectivity and Experimental Inhibition Mechanisms

Experimental kinetic characterization is available for only a small subset of plant-derived DPP-4 inhibitors (Table 5). Using human recombinant DPP-4, Pan et al. [35] showed that myricetin, hyperoside, narcissoside, cyanidin 3-O-glucoside, and isoliquiritigenin inhibited the enzyme reversibly. Myricetin displayed non-competitive inhibition, whereas the other four compounds showed mixed-type inhibition. For the mixed inhibitors, Kic was lower than Kiu, indicating a larger competitive contribution under the reported assay conditions. In an independent human recombinant DPP-4 system, chalconaringenin 2′-O-β-D-glucopyranoside and aureusidin 6-O-β-D-glucopyranoside also showed mixed competitive/non-competitive inhibition by Lineweaver–Burk analysis, although numerical Ki values were not reported [46].

By contrast, the cited primary studies did not report compound-specific inhibition of DPP-8 or DPP-9 for the kinetically characterized compounds in Table 5 or for the five priority scaffolds in Table 4. These entries are therefore recorded as not reported rather than interpreted as evidence of selectivity. Until head-to-head testing against recombinant human DPP-4, DPP-8, and DPP-9 is performed under matched conditions, therapeutic selectivity remains unestablished and the compounds should be regarded as early lead-like scaffolds [54,55,56].

## 8. Limitations and Opportunities

The major limitation of the natural DPP-4 inhibitor literature is the lack of cross-study comparability. Differences in DPP-4 source, substrate, buffer composition, concentration range, and detection method mean that IC_50_ values cannot be treated as directly comparable across studies [34,35,36]. Interpretation is further complicated when activity is reported for crude extracts, incompletely characterized fractions, or mixtures whose active constituents are uncertain. Matrix effects and chemical or metabolic stability under biologically relevant conditions also influence the likelihood that enzyme-level activity will translate beyond simplified assays [34,35].

The PAINS-related assay-interference concerns introduced above have direct implications for candidate prioritization. For polyphenolic compounds, activity should be reconfirmed using orthogonal assay formats and, where feasible, detergent, protein, or optical-interference controls to distinguish target-specific DPP-4 inhibition from colloidal aggregation, nonspecific protein binding, optical effects, or other physicochemical artifacts [37,38].

Selectivity is equally important. Clinically viable DPP-4 inhibitors should preferentially inhibit DPP-4 while avoiding functionally related proteases such as DPP-8 and DPP-9. Therefore, studies reporting only a single DPP-4 IC_50_ value without selectivity data should be interpreted as preliminary screening evidence rather than as sufficient justification for preclinical advancement [54,55].

Beyond assay standardization, selectivity, and compound stability, several additional translational considerations remain insufficiently addressed in the current literature. These include tissue distribution and the relative contribution of soluble versus membrane-associated DPP-4. The current review did not specifically assess these aspects, although they may influence local inhibitor exposure, pharmacodynamic activity, and interpretation of effects outside the circulating compartment [57].

As a result, the potential areas of advancement in this area for the near term should reduce the amount of time spent evaluating novel sets of fragments based upon screening studies and spend more time optimizing those leads that have been previously discovered. A logical course of action would involve repeating all assessments of DPP-4 inhibitory activity in human recombinant DPP-4, utilizing identical conditions throughout all experiments, comparing selectivity toward DPP-8 and DPP-9, validating inhibition through orthogonal methodologies, and moving from simple enzyme-based assays toward more physiologic cellular models and ultimately toward pre-clinical models. Only then will researchers be able to differentiate between highly promising lead scaffolds and compounds whose efficacy is dependent upon artifacts generated during assay procedures or instability of the active compound itself [35,56].

On a practical note, at this point, natural DPP-4 inhibitors should be viewed as primary resources for medicinal chemistry optimization of existing chemical scaffolds, rather than being nearly ready phytopharmaceutical products. Rather than expanding lists of “active” plants, researchers should seek to thoroughly verify a limited number of candidates which represent some of the best molecules identified to date, and then utilize targeted modifications to enhance their specificity, stability and pharmacological profiles. Making this transition from descriptive activity to standardized lead optimization represents exactly what will need to occur if this field is to develop new DPP-4 inhibitors effectively [36,57].

## 9. Conclusions

The review illustrates that while research into plant-derived DPP-4 inhibitors has moved past descriptive reporting to provide a number of useful avenues for development in the future, a large portion of the empirical evidence collected to date has focused on flavonoids and polyphenolic compounds. Nevertheless, the quality of evidence associated with these groups is generally low and comparisons among results are limited due to variability in experimental conditions including test systems, medium compositions and extent of experimental verification [35,36].

Critical examination suggests that future development should focus on a limited subset of chemically defined compounds supported by interpretable assay systems, quantitative DPP-4 inhibition data, and scaffolds amenable to further optimization. Within the available literature, chalcones and aurones are among the more consistently supported lead-like scaffolds, although the available evidence remains insufficient to establish therapeutic superiority. In particular, lanceoletin, leptosidin, xanthoangelol, chalconaringenin 2′-O-β-D-glucopyranoside, and aureusidin 6-O-β-D-glucopyranoside warrant further standardized evaluation. The berberine framework provides a chemically distinct alternative direction for expanding the search space beyond polyphenolic scaffolds [43,44].

At present the empirical data cannot be used to support use of plant-derived DPP-4 inhibitors as alternatives to commercially accepted antidiabetic medications. Their greatest utility is as an expansion of available chemical space for medicinal chemists and as platforms for identifying lead-like scaffolding that can serve as bases for further refinement/optimization. For the next stages of research key objectives will include standardized reassessment of potency/activity, evaluation of selectivity vs. DPP-8 and DPP-9, kinetic analyses, and progression to cellular and/or more physiologically relevant preclinical models [34,55,56].

Thus, in conclusion to this review, plant-derived DPP-4 inhibitors should be viewed primarily as a source of chemical scaffolds for further rational development rather than as an established therapeutic class. A major priority for this field is to progress from simply accumulating scattered reports of activity signals toward establishing an even tighter scrutiny system for reviewing evidence, prioritizing candidate compounds, and refining selections of candidates using medicinal chemistry techniques [35,36].

## Figures and Tables

**Figure 1:**
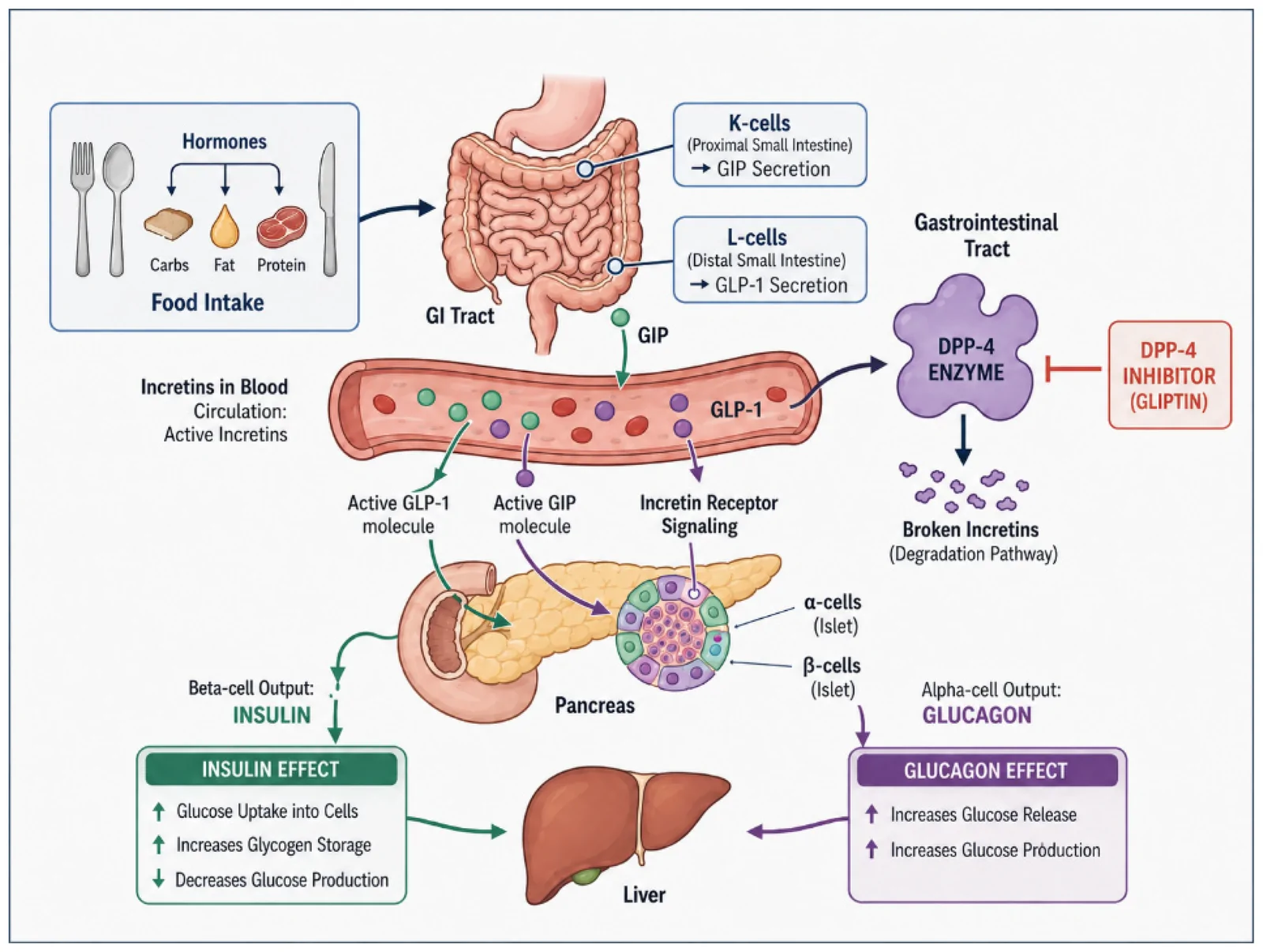
Schematic representation of the incretin axis and the principal metabolic effects of pharmacological DPP-4 inhibition. DPP-4 blockade prolongs endogenous GLP-1 and GIP activity, thereby enhancing glucose-dependent insulin secretion and suppressing glucagon release.

**Figure 2:**
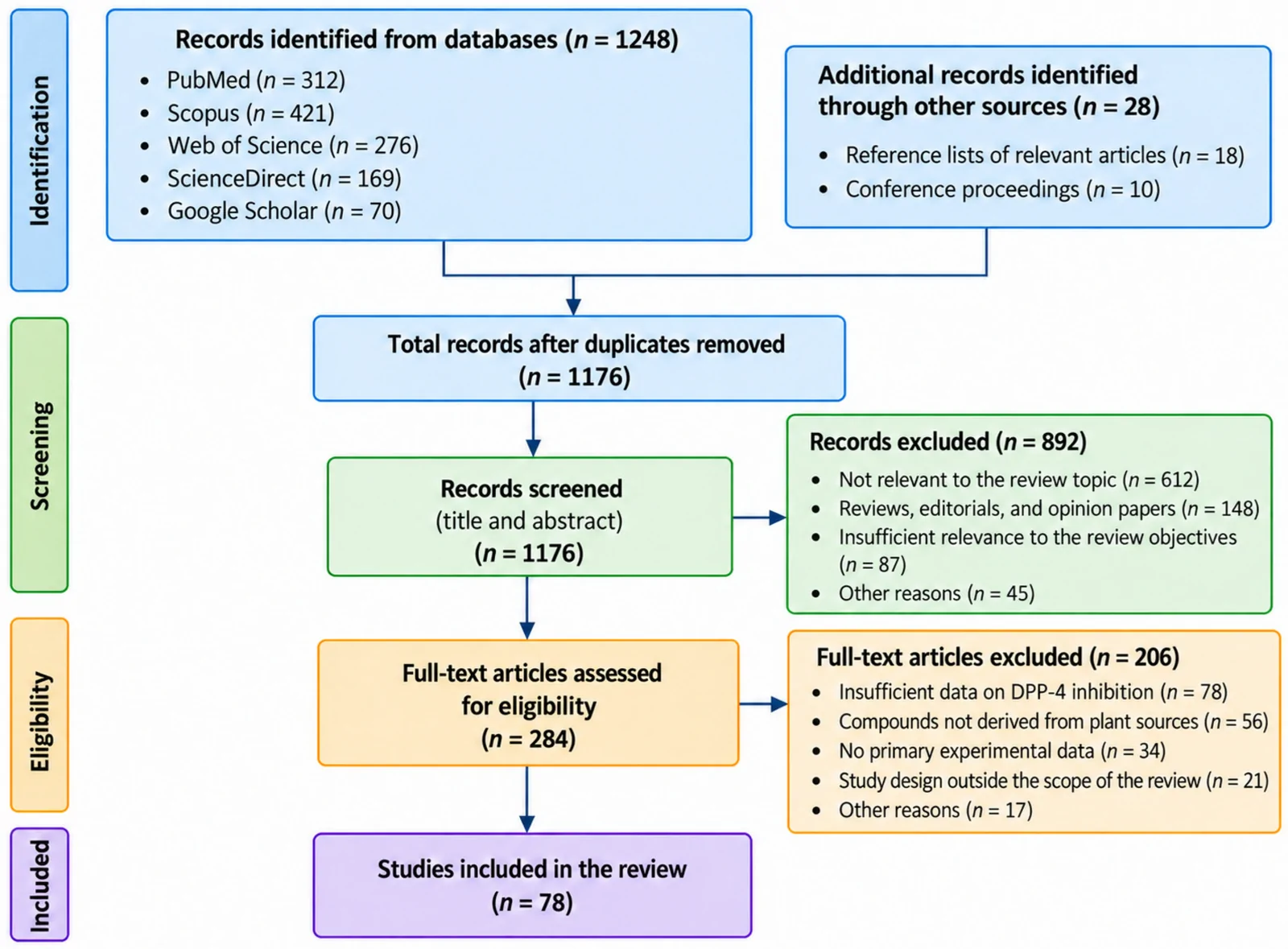
Flow diagram of the literature identification, screening, eligibility assessment, and study-selection process.

**Table 1:** Commercially Approved DPP-4 Inhibitors.

#	DPP-4 Inhibitor	Brand Name	Approval Year
1	Sitagliptin	Januvia	2006 [12]
2	Saxagliptin	Onglyza	2009 [13]
3	Linagliptin	Tradjenta	2011 [14]
4	Alogliptin	Nesina	2013 [15]
5	Gemigliptin	Zemiglo	2012 [16]
6	Evogliptin	Suganon	2015 [17]
7	Teneligliptin	Tenelia	2012 [18]
8	Anagliptin	Suiny	2012 [19]
9	Trelagliptin	Zafatek	2015 [20]
10	Omarigliptin	Marizev	2015 [21]

**Table 2:** Combination Products Containing DPP-4 Inhibitors.

#	Combination	Brand Name	Approval Year
1	Sitagliptin + metformin	Janumet	2007 [22]
2	Saxagliptin + metformin XR	Kombiglyze XR	2010 [23]
3	Linagliptin + metformin	Jentadueto	2012 [24]
4	Vildagliptin + metformin	Eucreas (EU)	2007 [25]
5	Alogliptin + metformin	Kazano	2013 [26]
6	Alogliptin + pioglitazone	Oseni	2013 [27]
7	Linagliptin + empagliflozin	Glyxambi	2015 [28]
8	Dapagliflozin + saxagliptin	Qtern	2017 [29]
9	Ertugliflozin + sitagliptin	Steglujan	2017 [30]
10	Empagliflozin + linagliptin + metformin XR	Trijardy XR	2020 [31]

**Table 3:** Representative plant-derived DPP-4 inhibitors selected for critical comparison.

№	Plant Natural Compound	Class	Reported IC_50_ (as Published)	DPP-4 Source	Assay System/Substrate	Plant Source (Exact Species, Part)	Reference
1	Kaempferol-3-O-β-glucopyranosyl-(1→2)-β-galactopyranosyl-7-O-α-rhamnopyranoside	Flavonol glycoside	27.89 ± 1.29 µM	Human recombinant DPP-4	Cayman fluorescence assay; Gly-Pro-AMC substrate; Ex 350–360/Em 450–465 nm	*Lens culinaris* Medikus, seeds	[39]
9	Chalconaringenin 2′-O-β-D-glucopyranoside	Chalcone glycoside	23.1 µM	Human recombinant DPP-4	Fluorescence microplate assay; Gly-Pro-AMC substrate; Ex 380/Em 460 nm	*Helichrysum arenarium* L. Moench, flowers	[46]
10	Chalconaringenin 2′,4′-di-O-β-D-glucopyranoside	Chalcone diglycoside	70.0 µM	Human recombinant DPP-4	Fluorescence microplate assay; Gly-Pro-AMC substrate; Ex 380/Em 460 nm	*Helichrysum arenarium* L. Moench, flowers	[46]
23	Aureusidin 6-O-β-D-glucopyranoside	Aurone glycoside	24.3 µM	Human recombinant DPP-4	Fluorescence microplate assay; Gly-Pro-AMC substrate; Ex 380/Em 460 nm	*Helichrysum arenarium* L. Moench, flowers	[46]
26	4-Hydroxyderricin	Chalcone	81.44 µM	Human DPP-4	Colorimetric 96-well assay; Gly-Pro-p-nitroanilide (GPPN) substrate; A405	*Angelica keiskei* Koidzumi, sap	[50]
27	Xanthoangelol	Chalcone	10.49 µM	Human DPP-4	Colorimetric DPP-4 assay; GPPN substrate; A405 (method of Aulifa et al. 2022) [48]	*Angelica keiskei* Koidzumi, yellow sap	[48]
28	Lanceoletin	Chalcone	9.6 µM	Human recombinant DPP-4	Cayman fluorescence assay; H-Gly-Pro-AMC substrate; Ex 350/Em 450 nm	*Coreopsis lanceolata* L., flower	[45]
29	3,2′-Dihydroxy-4,3′-dimethoxychalcone-4′-glucoside	Chalcone glycoside	14.3 µM	Human recombinant DPP-4	Cayman fluorescence assay; H-Gly-Pro-AMC substrate; Ex 350/Em 450 nm	*Coreopsis lanceolata* L., flower	[45]
30	4-Methoxylanceoletin	Chalcone	21.6 µM	Human recombinant DPP-4	Cayman fluorescence assay; H-Gly-Pro-AMC substrate; Ex 350/Em 450 nm	*Coreopsis lanceolata* L., flower	[45]
31	Leptosidin	Aurone	13.3 µM	Human recombinant DPP-4	Cayman fluorescence assay; H-Gly-Pro-AMC substrate; Ex 350/Em 450 nm	*Coreopsis lanceolata* L., flower	[45]
35	(−)-Vitisin B	Stilbenoid oligomer	15.3 µM	Porcine kidney DPP-4	Porcine kidney DPP-4 enzymatic assay; substrate/detection not explicitly detailed in accessible primary text	*Vitis thunbergii* var. *taiwaniana*, stem	[51]
36	Cyanidin 3,5-diglucoside	Anthocyanin diglycoside	5.5 mM	DPP-4 purified from porcine seminal plasma	Fluorometric assay; Gly-Pro-MCA substrate; porcine seminal-plasma DPP-4	Aronia juice, species not explicitly stated in the paper	[52]
39	O-Ethyl-4-[(α-l-rhamnosyloxy)-benzyl] carbamate	Carbamate glycoside	798 nM	Assay kit stated, enzyme origin not explicitly stated in the paper text	Abnova KA1311 fluorescence assay; substrate not explicitly stated in article text	*Moringa oleifera* Lam., seeds	[53]

Note: IC50 values are reproduced as reported in the original studies and are not normalized across experimental systems. They should therefore not be interpreted as a direct ranking of compound potency. Meaningful quantitative comparisons are primarily limited to compounds evaluated within the same study using the same enzyme source, substrate, assay format, and experimental conditions. The complete evidence map is provided in Supplementary Table S1.

**Table 4:** Priority plant-derived DPP-4 scaffolds for further investigation based on an integrated evidence assessment.

Compound	Class	Reported IC_50_	DPP-4 Source	Plant Source (Species, Part)	Rationale for Prioritization	Experimental Inhibition Mechanism/Molecular Evidence	DPP-8/DPP-9 Selectivity	Cellular/In Vivo Evidence	Translational Limitation
Lanceoletin [45]	Chalcone	9.6 µM	Human recombinant DPP-4	*Coreopsis lanceolata* L., flower	One of the strongest hits in a defined recombinant human assay; attractive lead scaffold for optimization	Experimental inhibition type and kinetic constants were not reported. Biochemical inhibition was demonstrated; compound-specific docking was not reported [45].	Not reported in the cited DPP-4 study.	No compound-specific cellular or in vivo DPP-4 validation reported.	No reported DPP-8/DPP-9 selectivity, cellular/in vivo validation, or pharmacokinetic characterization.
Xanthoangelol [48]	Chalcone	10.49 µM	Human DPP-4	*Angelica keiskei* Koidzumi, yellow sap	Low-micromolar activity with a chemically tractable chalcone framework	Experimental inhibition type and kinetic constants were not reported. Docking to human DPP-4 proposed H-bonds with Glu205/Glu206 and a π–π interaction with Phe357; in vitro activity was also demonstrated [48].	Not reported in the cited DPP-4 study.	No direct DPP-4 cellular or in vivo validation reported; the cited study was in vitro and in silico.	Docking remains predictive; DPP-8/DPP-9 selectivity and direct cellular/in vivo DPP-4 validation remain unestablished.
Leptosidin [45]	Aurone	13.3 µM	Human recombinant DPP-4	*Coreopsis lanceolata* L., flower	Strong activity and a structurally distinct aurone scaffold suitable for further lead optimization	Experimental inhibition type and kinetic constants were not reported. Biochemical inhibition was demonstrated; compound-specific docking was not reported [45].	Not reported in the cited DPP-4 study.	No compound-specific cellular or in vivo DPP-4 validation reported.	Limited mechanistic, selectivity, cellular/in vivo, and pharmacokinetic validation.
Chalconaringenin 2′-O-β-D-glucopyranoside [46]	Chalcone glycoside	23.1 µM	Human recombinant DPP-4	*Helichrysum arenarium* L. Moench, flowers	One of the most convincing *Helichrysum* leads and an important confirmation of the chalcone motif	Mixed competitive/non-competitive inhibition was demonstrated by Lineweaver-Burk analysis; numerical Ki values and compound-specific docking were not reported [46].	Not reported in the cited DPP-4 study.	No compound-specific cellular/in vivo DPP-4 validation; the parent *H. arenarium* extract showed antihyperglycemic activity in sucrose-loaded mice.	Compound-specific selectivity, cellular/in vivo DPP-4 validation, and pharmacokinetic evidence remain limited.
Aureusidin 6-O-β-D-glucopyranoside [46]	Aurone glycoside	24.3 µM	Human recombinant DPP-4	*Helichrysum arenarium* L. Moench, flowers	A promising aurone-type scaffold with favorable activity and clear medicinal-chemistry relevance	Mixed competitive/non-competitive inhibition was demonstrated by Lineweaver-Burk analysis; numerical Ki values and compound-specific docking were not reported [46].	Not reported in the cited DPP-4 study.	No compound-specific cellular/in vivo DPP-4 validation; the parent *H. arenarium* extract showed antihyperglycemic activity in sucrose-loaded mice.	Compound-specific selectivity, cellular/in vivo DPP-4 validation, and pharmacokinetic evidence remain limited.

Note: prioritization integrates assay quality, within-series potency, mechanistic evidence where available, and scaffold tractability. Docking results are hypothesis-generating and are not equivalent to experimental binding data. Absence of reported DPP-8/DPP-9 selectivity or cellular/in vivo validation should not be interpreted as evidence that these properties have been established. Where ‘Not reported’ is used, it refers specifically to information not reported for the individual compound in the cited DPP-4 study; evidence from a parent extract is stated separately. Reported IC50 values in this table originate from different experimental studies and should not be compared as a standardized potency ranking. Prioritization reflects the integrated evidence assessment described in Section 7.3 rather than IC50 alone.

**Table 5:** Experimentally characterized inhibition mechanisms and reported DPP-8/DPP-9 selectivity.

Compound	Experimental Inhibition Mode	Reported Kinetic Parameters	DPP-8/DPP-9 Selectivity	Reference
Hyperoside	Reversible, mixed-type	Kic 90.3 ± 1.0 µM; Kiu 148 ± 2.2 µM	Not reported in the cited primary study	[35]
Myricetin	Reversible, non-competitive	Kic = Kiu = 103.5 ± 1.1 µM	Not reported in the cited primary study	[35]
Narcissoside	Reversible, mixed-type	Kic 115.7 ± 2.1 µM; Kiu 223 ± 1.5 µM	Not reported in the cited primary study	[35]
Cyanidin 3-O-glucoside	Reversible, mixed-type	Kic 74.3 ± 1.2 µM; Kiu 183.7 ± 2.4 µM	Not reported in the cited primary study	[35]
Isoliquiritigenin	Reversible, mixed-type	Kic 98.2 ± 3.4 µM; Kiu 267 ± 3.4 µM	Not reported in the cited primary study	[35]
Chalconaringenin 2′-O-β-D-glucopyranoside	Mixed competitive/non-competitive	Numerical Ki values not reported	Not reported in the cited primary study	[46]
Aureusidin 6-O-β-D-glucopyranoside	Mixed competitive/non-competitive	Numerical Ki values not reported	Not reported in the cited primary study	[46]

Note: Kic and Kiu values from Pan et al. [35] were converted from 10−5 mol/L to micromolar units. Inhibition modes were assigned experimentally using enzyme-rate plots, Lineweaver–Burk analysis, and, for the Pan et al. series, Dixon and Cornish-Bowden plots. ‘Not reported’ indicates that the cited primary study did not provide the corresponding compound-specific DPP-8/DPP-9 data; it does not establish selectivity.

## Data Availability

No new data were created or analyzed in this study. Data sharing is not applicable to this article.

## Supplementary Materials

The following supporting information can be downloaded at https://www.mdpi.com/article/10.3390/biomedicines14102141/s1, Supplementary Table S1: Full curated catalogue of plant compounds with reported DPP-4 inhibitory activity.
