# Supplementary material for: Plant-Derived Dipeptidyl Peptidase-4 Inhibitors in Cardiometabolic Disorders: A Critical Review of Natural Scaffolds and Experimental Evidence
Source: Biomedicines. 2026 Sep 22;14(10):2141. doi: 10.3390/biomedicines14102141 (PMC13701426; doi:10.3390/biomedicines14102141)
Supplement: Supplementary file 1 [file biomedicines-14-02141-s001.zip › biomedicines-4542720-Supplementary.pdf]

Supplementary Table S1. Full curated catalogue of plant compounds with reported DPP-4 inhibitory activity

IC50 values and assay characteristics are reproduced from the cited primary literature. Values are not normalized across experimental systems and should not be interpreted as a direct potency ranking. Quantitative comparisons are most defensible for compounds tested within the same study using the same enzyme source, substrate, assay format, and experimental conditions. Where assay details were not explicitly available in the primary article text, this is stated directly in the table.

№	Plant natural compound	Class	Reported IC_{50} (as published)	DPP-4 source	Assay system / substrate	Plant source (exact species, part)	Ref.
1	Kaempferol-3-O-β-glucopyranosyl-(1→2)-β-galactopyranosyl-7-O-α-rhamnopyranoside	Flavonol glycoside	27.89 ± 1.29 μM	Human recombinant DPP-4	Cayman fluorescence assay; Gly-Pro-AMC substrate; Ex 350–360/Em 450–465 nm	*Lens culinaris* Medikus, seeds	[39]
2	Kaempferol-3-O-β-glucopyranosyl-(1→2)-[α-rhamnopyranosyl(1→6)]-β-galactopyranosyl-7-O-α-rhamnopyranoside	Flavonol glycoside	36.52 ± 0.78 μM	Human recombinant DPP-4	Cayman fluorescence assay; Gly-Pro-AMC substrate; Ex 350–360/Em 450–465 nm	*Lens culinaris* Medikus, seeds	[39]
3	Robinin	Flavonol glycoside	37.01 ± 1.40 μM	Human recombinant DPP-4	Cayman fluorescence assay; Gly-Pro-AMC substrate; Ex 350–360/Em 450–465 nm	*Lens culinaris* Medikus, seeds	[39]
4	Methyl p-coumarate	Phenylpropanoid ester	911.44 ± 7.61 μM	Human recombinant DPP-4	Cayman fluorescence assay; Gly-Pro-AMC substrate; Ex 350–360/Em 450–465 nm	*Melicope latifolia* (DC.) T.G. Hartley, bark	[40]
5	Arenariumoside V	Dimeric dihydrochalcone glycoside	83.0 μM	Human recombinant DPP-4	Fluorescence microplate assay; Gly-Pro-AMC substrate; Ex 380/Em 460 nm	*Helichrysum arenarium* L. Moench, flowers	[46]
6	Arenariumoside VI	Dimeric dihydrochalcone glycoside	52.0 μM	Human recombinant DPP-4	Fluorescence microplate assay; Gly-Pro-AMC substrate; Ex 380/Em 460 nm	*Helichrysum arenarium* L. Moench, flowers	[46]
7	Arenariumoside VII	Dimeric dihydrochalcone glycoside	83.0 μM	Human recombinant DPP-4	Fluorescence microplate assay; Gly-Pro-AMC substrate; Ex 380/Em 460 nm	*Helichrysum arenarium* L. Moench, flowers	[46]
8	Arenariumoside III	Dimeric dihydrochalcone glycoside	54.1 μM	Human recombinant DPP-4	Fluorescence microplate assay; Gly-Pro-AMC substrate; Ex 380/Em 460 nm	*Helichrysum arenarium* L. Moench, flowers	[46]
9	Chalconaringenin 2′-O-β-D-glucopyranoside	Chalcone glycoside	23.1 μM	Human recombinant DPP-4	Fluorescence microplate assay; Gly-Pro-AMC substrate; Ex 380/Em 460 nm	*Helichrysum arenarium* L. Moench, flowers	[46]
10	Chalconaringenin 2′,4′-di-O-β-D-glucopyranoside	Chalcone diglycoside	70.0 μM	Human recombinant DPP-4	Fluorescence microplate assay; Gly-Pro-AMC substrate; Ex 380/Em 460 nm	*Helichrysum arenarium* L. Moench, flowers	[46]
11	Apigenin 7-O-β-D-glucopyranoside	Flavone glycoside	62.7 μM	Human recombinant DPP-4	Fluorescence microplate assay; Gly-Pro-AMC substrate; Ex 380/Em 460 nm	*Helichrysum arenarium* L. Moench, flowers	[46]
12	Apigenin 7-O-β-D-glucopyranosiduronic acid methyl ester	Flavone glycoside	50.5 μM	Human recombinant DPP-4	Fluorescence microplate assay; Gly-Pro-AMC substrate; Ex 380/Em 460 nm	*Helichrysum arenarium* L. Moench, flowers	[46]
13	Apigenin 7-O-gentiobioside	Flavone diglycoside	32.0 μM	Human recombinant DPP-4	Fluorescence microplate assay; Gly-Pro-AMC substrate; Ex 380/Em 460 nm	*Helichrysum arenarium* L. Moench, flowers	[46]
14	Luteolin 7-O-β-D-glucopyranoside	Flavone glycoside	37.5 μM	Human recombinant DPP-4	Fluorescence microplate assay; Gly-Pro-AMC substrate; Ex 380/Em 460 nm	*Helichrysum arenarium* L. Moench, flowers	[46]
15	6-Hydroxyluteolin 7-O-β-D-glucopyranoside	Flavone glycoside	60.3 μM	Human recombinant DPP-4	Fluorescence microplate assay; Gly-Pro-AMC substrate; Ex 380/Em 460 nm	*Helichrysum arenarium* L. Moench, flowers	[46]
16	6-Hydroxy-3′-O-methylluteolin 7-O-β-D-glucopyranoside	O-methylated flavone glycoside	73.2 μM	Human recombinant DPP-4	Fluorescence microplate assay; Gly-Pro-AMC substrate; Ex 380/Em 460 nm	*Helichrysum arenarium* L. Moench, flowers	[46]
17	Kaempferol 3-O-gentiobioside	Flavonol diglycoside	58.0 μM	Human recombinant DPP-4	Fluorescence microplate assay; Gly-Pro-AMC substrate; Ex 380/Em 460 nm	*Helichrysum arenarium* L. Moench, flowers	[46]
18	Kaempferol 3,7-di-O-β-D-glucopyranoside	Flavonol diglycoside	36.8 μM	Human recombinant DPP-4	Fluorescence microplate assay; Gly-Pro-AMC substrate; Ex 380/Em 460 nm	*Helichrysum arenarium* L. Moench, flowers	[46]
19	Kaempferol 3,4′-di-O-β-D-glucopyranoside	Flavonol diglycoside	88.6 μM	Human recombinant DPP-4	Fluorescence microplate assay; Gly-Pro-AMC substrate; Ex 380/Em 460 nm	*Helichrysum arenarium* L. Moench, flowers	[46]
20	Kaempferol 3-O-β-D-glucopyranosyl-(1→3)-β-D-glucopyranoside	Flavonol diglycoside	85.2 μM	Human recombinant DPP-4	Fluorescence microplate assay; Gly-Pro-AMC substrate; Ex 380/Em 460 nm	*Helichrysum arenarium* L. Moench, flowers	[46]
21	Quercetin 3-O-β-D-glucopyranoside	Flavonol glycoside	56.6 μM	Human recombinant DPP-4	Fluorescence microplate assay; Gly-Pro-AMC substrate; Ex 380/Em 460 nm	*Helichrysum arenarium* L. Moench, flowers	[46]
22	Quercetin 3,3′-di-O-β-D-glucopyranoside	Flavonol diglycoside	73.9 μM	Human recombinant DPP-4	Fluorescence microplate assay; Gly-Pro-AMC substrate; Ex 380/Em 460 nm	*Helichrysum arenarium* L. Moench, flowers	[46]
23	Aureusidin 6-O-β-D-glucopyranoside	Aurone glycoside	24.3 μM	Human recombinant DPP-4	Fluorescence microplate assay; Gly-Pro-AMC substrate; Ex 380/Em 460 nm	*Helichrysum arenarium* L. Moench, flowers	[46]
24	3,6′-O-diferuloylsucrose	Phenylpropanoid sucrose ester	46.19 μM	Human recombinant DPP-4	Cayman fluorescence assay; H-Gly-Pro-AMC substrate; Ex 350/Em 450 nm	*Lilium longiflorum* hybrid cv. White Triumph, scaly bulbs	[41]
25	4-O-acetyl-3,6′-O-diferuloylsucrose	Phenylpropanoid sucrose ester	63.26 μM	Human recombinant DPP-4	Cayman fluorescence assay; H-Gly-Pro-AMC substrate; Ex 350/Em 450 nm	*Lilium longiflorum* hybrid cv. White Triumph, scaly bulbs	[41]

№	Plant natural compound	Class	Reported IC_{50} (as published)	DPP-4 source	Assay system / substrate	Plant source (exact species, part)	Ref.
26	4-Hydroxyderricin	Chalcone	81.44 μM	Human DPP-4	Colorimetric 96-well assay; Gly-Pro-p-nitroanilide (GPPN) substrate; A405	*Angelica keiskei* Koidzumi, sap	[50]
27	Xanthoangelol	Chalcone	10.49 μM	Human DPP-4	Colorimetric DPP-4 assay; GPPN substrate; A405 (method of Aulifa et al. 2019)	*Angelica keiskei* Koidzumi, yellow sap	[48]
28	Lanceoletin	Chalcone	9.6 μM	Human recombinant DPP-4	Cayman fluorescence assay; H-Gly-Pro-AMC substrate; Ex 350/Em 450 nm	*Coreopsis lanceolata* L., flower	[45]
29	3,2′-Dihydroxy-4,3′-dimethoxychalcone-4′-glucoside	Chalcone glycoside	14.3 μM	Human recombinant DPP-4	Cayman fluorescence assay; H-Gly-Pro-AMC substrate; Ex 350/Em 450 nm	*Coreopsis lanceolata* L., flower	[45]
30	4-Methoxylanceoletin	Chalcone	21.6 μM	Human recombinant DPP-4	Cayman fluorescence assay; H-Gly-Pro-AMC substrate; Ex 350/Em 450 nm	*Coreopsis lanceolata* L., flower	[45]
31	Leptosidin	Aurone	13.3 μM	Human recombinant DPP-4	Cayman fluorescence assay; H-Gly-Pro-AMC substrate; Ex 350/Em 450 nm	*Coreopsis lanceolata* L., flower	[45]
32	(2R)-8-Methoxybutin	Flavanone	64.9 μM	Human recombinant DPP-4	Cayman fluorescence assay; H-Gly-Pro-AMC substrate; Ex 350/Em 450 nm	*Coreopsis lanceolata* L., flower	[45]
33	(+)-Hopeaphenol	Stilbenoid oligomer	401 μM	Porcine kidney DPP-4	Porcine kidney DPP-4 enzymatic assay; substrate/detection not explicitly detailed in accessible primary text	*Vitis thunbergii* var. *taiwaniana*, stem	[51]
34	(+)-Vitisin A	Stilbenoid oligomer	90.75 μM	Porcine kidney DPP-4	Porcine kidney DPP-4 enzymatic assay; substrate/detection not explicitly detailed in accessible primary text	*Vitis thunbergii* var. *taiwaniana*, stem	[51]
35	(−)-Vitisin B	Stilbenoid oligomer	15.3 μM	Porcine kidney DPP-4	Porcine kidney DPP-4 enzymatic assay; substrate/detection not explicitly detailed in accessible primary text	*Vitis thunbergii* var. *taiwaniana*, stem	[51]
36	Cyanidin 3,5-diglucoside	Anthocyanin diglycoside	5.5 mM	DPP-4 purified from porcine seminal plasma	Fluorometric assay; Gly-Pro-MCA substrate; porcine seminal-plasma DPP-4	Aronia juice, species not explicitly stated in the paper	[52]
37	Rugosin A	Ellagitannin	25.8 μM	Not explicitly stated in the accessible paper text	Cayman DPP-4 screening kit; fluorescence Ex 360/Em 460 nm; substrate not named in article text	*Rosa gallica* L., flower buds	[42]
38	Rugosin B	Ellagitannin	28.5 μM	Not explicitly stated in the accessible paper text	Cayman DPP-4 screening kit; fluorescence Ex 360/Em 460 nm; substrate not named in article text	*Rosa gallica* L., flower buds	[42]
39	O-Ethyl-4-[(α-l-rhamnosyloxy)-benzyl] carbamate	Carbamate glycoside	798 nM	Assay kit stated, enzyme origin not explicitly stated in the paper text	Abnova KA1311 fluorescence assay; substrate not explicitly stated in article text	*Moringa oleifera* Lam., seeds	[53]
